# COVID-19 epidemiology, health services utilisation and health care seeking behaviour during the first year of the COVID-19 pandemic in Mweso health zone, Democratic Republic of Congo

**DOI:** 10.7189/jogh.14.05016

**Published:** 2024-04-26

**Authors:** Chiara Altare, Natalya Kostandova, Linda Matadi Basadia, Marie Petry, Gbètoho Fortuné Gankpe, Hannah Crockett, Natalia Hernandez Morfin, Sophie Bruneau, Caroline Antoine, Paul B Spiegel, IMPACT DRC Team, Roxana Mullafiroze, Jasper Linke, Olivier Cecchi, Nayana Das, Katie Rickard, Jean-Paul Mushamalirwa, Destin Ruhinda, Nadia Lehmann, Marie Amandine, Eliora Henzler, Audrey Gallecier, Benoit Besnardeau, Noortje Gerritsma

**Affiliations:** 1 Department of International Health, Johns Hopkins Bloomberg School of Public Health, Baltimore, Maryland, USA; 2 Johns Hopkins Center for Humanitarian Health, Baltimore, Maryland, USA; 3 Health and Nutrition Department, Action Contre la Faim, Kinshasa, Democratic Republic of Congo; 4 Operations Department, Action Contre la Faim, Paris, France; 5 Technical and Advocacy Department, Action Contre la Faim, Paris, France

## Abstract

**Background:**

Although the evidence about coronavirus disease 2019 (COVID-19) has increased exponentially since the beginning of the pandemic, less is known about the direct and indirect effects of the pandemic in humanitarian settings. In the Democratic Republic of the Congo (DRC), most studies occurred in Kinshasa and other cities. Limited research was conducted in remote conflict-affected settings. We investigated the COVID-19 epidemiology, health service utilisation, and health care-seeking behaviour during the first year of the pandemic (March 2020–March 2021) in the Mweso health zone, North Kivu, DRC.

**Methods:**

This mixed-methods study includes a descriptive epidemiological analysis of reported COVID-19 cases data extracted from the provincial line list, interrupted time series analysis of health service utilisation using routine health service data, qualitative perceptions of health care workers about how health services were affected, and community members’ health care seeking behaviour from a representative household survey and focus group discussions.

**Results:**

The COVID-19 epidemiology in North Kivu aligns with evidence reported globally, yet case fatality rates were high due to underreporting. Testing capacity was limited and initially mainly available in the province’s capital. Health service utilisation showed different patterns – child measles vaccinations experienced a decrease at the beginning of the pandemic, while outpatient consultations, malaria, and pneumonia showed an increase over time. Such increases might have been driven by insecurity and population displacements rather than COVID-19. Community members continued seeking care during the first months of the COVID-19 pandemic and visited the same health facilities as before COVID-19. Financial constraints, not COVID-19, were the main barrier reported to accessing health care.

**Conclusions:**

The first year of the COVID-19 pandemic in the Mweso health zone was characterised by low testing capacity and an underestimation of reported COVID-19 infections. The increase in health care utilisation should be further explored to understand the role of factors unrelated to COVID-19, such as insecurity, population displacement, and poverty, which remain major challenges to successfully providing health services and improving the population’s health. Measles vaccination coverage dropped, which exacerbated the ongoing measles outbreak. Improved decentralised testing capacity will be crucial for future epidemics and enhanced efforts to maintain child vaccination coverage.

Since its emergence in December 2019 in China, the severe acute respiratory syndrome coronavirus 2 (SARS-CoV-2) virus has affected all countries and all aspects of our societies. Despite the numerous governmental response strategies aiming to contain the spread of the disease, the world reached more than 643 million cases and 6.6 million deaths by December 2022 [1]. Health systems in nearly all countries were disrupted regarding the management of coronavirus disease 2019 (COVID-19) cases and the efforts to maintain routine health care services.

Initial assumptions expected low- and middle-income countries (LMICs) and humanitarian settings to be the least prepared to respond [2], given their already fragile health systems with limited human and financial resources, few existing intense care units, precarious population health status, poverty, and substandard water and sanitation conditions [3]. Early modelling studies aiming at estimating the burden of infections in LMICs and forced displacement settings suggested quite worrisome scenarios [4,5]. While several waves were recorded in LMICs and humanitarian settings, the dire forecasts did not seem to have occurred at the expected level, likely due to a mix of lower testing rates, a high proportion of asymptomatic cases, and a low proportion of cases experiencing severe outcomes and death [6,7]. Yet, as of December 2020, there were 20 million confirmed cases and 389 000 deaths reported in humanitarian emergencies in LMICs [8].

How countries were affected varied due to several factors, including the timely establishment of public health and social measures, existing response capacity to epidemics and emergencies, and demographic factors. The indirect effects of the pandemic on the health system’s capacity to maintain essential services were of similar or greater concern. Learning from large epidemics such as Ebola in West Africa and cholera in Yemen [9,10], governments wanted to avoid excess morbidity and mortality from infectious and chronic diseases, usually addressed with routine care. While national authorities and other health actors quickly recognised the need to adapt service delivery to reduce the risk of infection and ensure service continuity, challenges were extensive. Financial and human resources were limited and focused on COVID-19, while procurement chains were disrupted worldwide.

Knowledge and understanding about the virus’ behaviour have greatly increased since early 2020, yet few studies have been conducted in humanitarian settings. In the Democratic Republic of Congo (DRC), most studies occurred at the national level or in the capital, Kinshasa, and other major cities such as Lubumbashi, Bukavu, and Goma. These studies encompassed a broad range of topics, from serosurveys in cities such as Kinshasa, Goma and Bukavu [11–13], various waves of infection and actors’ response capacities [14,15], infection prevention and control and preventive interventions in internally displaced persons communities [16,17], broader effects of the pandemic on quality of life and livelihoods [18,19], synergies between COVID-19 and Ebola response strategies [20–22], COVID-19 knowledge, attitude and practice [23–25], vaccine acceptance [26–28], and investigations of hospitalised COVID-19 patients [29–31]. Healthcare utilisation was examined in Kinshasa [32,33] and at the national level [34–36]. Remote and hard-to-reach conflict-affected settings in North Kivu, Eastern DRC, remain less studied. We are aware of one study focusing on utilising sexual and reproductive health services in North Kivu province [37] and one in Goma [38].

Our study focused on the first year of the COVID-19 pandemic in Mweso health zone, North Kivu, DRC, and aimed to report on the epidemiology of COVID-19 and investigate how health service utilisation and health care-seeking behaviour have changed. This article is one of the three case studies [39–41] in humanitarian and fragile settings conducted within the United States Agency for International Development funded collaboration between the Johns Hopkins Centre for Humanitarian Health, Action Contre la Faim, and IMPACT. The other two case studies focus on Central African Republic and Cox’s Bazar, Bangladesh.

## METHODS

### Study setting

The study was implemented in Mweso health zone, North Kivu, DRC. North Kivu has suffered from decades of conflict and insecurity between numerous armed groups and the national defence forces [42], which have resulted in cross-border refugee movement and numerous bouts of internal displacement, as well as violent events that limited humanitarian access, particularly in the Mweso health zone [43]. Action Contre la Faim, one of the study partners, was one of the few humanitarian organisations active in the area. Mweso health zone is mainly rural and hosts a population of about 450 000, with one main town (Mweso). It includes 22 health areas (i.e. subdivisions within the health zone), each with a health centre and one reference hospital (Mweso).

### Data sources and study outcomes

This mixed-methods study has four components using four data sources.

The first component included the COVID-19 data. Confirmed COVID-19 cases were recorded on the provincial COVID-19 line list between 27 March 2020 (the first case reported in North Kivu) and 31 March 2021. Individual-level variables included patient demographic information, location, test data, exposure risks, case management, and disease outcome (Section 1.1 and Table S1 in the **Online Supplementary Document**). Testing data included the weekly number of conducted tests and related results in North Kivu from 1 June 2020–31 March 2021. National-level data (tests and cases) originated from the Johns Hopkins COVID-19 Resource Centre and Our World in Data [1,44].

The second component included routine health data. The monthly number of new outpatient consultations, consultations for suspected malaria and mild pneumonia, and first antenatal care visits was extracted from the National Health Information System. The number of monthly measles vaccine doses delivered originated from the District Vaccination Data Management Tool, and the weekly number of suspected measles and cholera cases from the epidemic-prone disease surveillance system. The study covers the period from 1 January 2017–31 March 2021. Outcomes of interest included health utilisation rate, rate of consultations for infectious diseases (malaria and pneumonia), measles cases, cholera cases, first antenatal care visit coverage, and measles vaccination coverage (Table S2 in the **Online Supplementary Document**). All data were aggregated at the health area level, and authors did not have access to identifiable information.

The third component included qualitative interviews with health care workers (HCWs). We interviewed 39 HCWs between March and June 2021 in 13 health facilities of various levels (nine health centres, two referral health centres, one health post, and one hospital). Professionals in different roles were included to comprise various perceptions (50% were nurses, followed by midwives, nutritionists, and pharmacists) (Table S3 in the **Online Supplementary Document**). Interviews were conducted in French, and answers were recorded on the interview guide.

Finally, the fourth component included communities’ perspectives on health care-seeking behaviour during the pandemic. Primary quantitative data were collected by IMPACT via a two-stage cluster sampling household survey (4–13 November 2021). From a list of 148 settlements larger than five hectares, 28 settlements were randomly selected, and households were identified using a randomly selected global positioning system point within the boundaries of the settlement. At least 12 households were interviewed per settlement. Out of the 28 settlements, six were not accessible and were not replaced due to logistical and security constraints. The corresponding number of interviews took place in accessible settlements nearby. A sample size of 657 persons was calculated with a 5% margin of error, 95% confidence interval (CI), and an intra-cluster correlation of 0.06. Furthermore, we conducted 12 semi-structured focus group discussions with 110 Swahili-speaking participants, including community and religious leaders, the elderly, business owners, and other community members (Table S4 in the **Online Supplementary Document**). Focus group participants were recruited by IMPACT and Action Contre la Faim teams a few days before the focus groups. Qualitative data were collected between 27 October and 2 November 2021. The map in Figure S1 in the **Online Supplementary Document** shows locations assessed through household surveys and focus group discussions and inaccessible areas due to security constraints.

### Analytical approach

#### COVID-19 epidemiology

We performed a descriptive analysis to calculate the number of cumulative cases, testing and incidence rates, age and gender distribution, and clinical presentation. We used multivariable logistic regression to assess risk factors associated with disease mortality (age, sex, residence). We checked the assumptions and model fit of the logistic regression (Section 2.1.3 in the **Online Supplementary Document**). We considered *P*-values <0.05 as statistically significant. Analyses were conducted in Stata, version 14 (StataCorp LLC, College Station, TX, USA).

#### Changes in health service utilisation

Mweso health zone’s 22 health areas were regrouped into five supervision subregions (aligning with health organisations’ programming) – Central, Kitshanga, Mokoto, Kirumbu, Bibwe (Table S5 in the **Online Supplementary Document**). Outliers from the pre-COVID-19 period (i.e. observations ≥3 standard deviations away from the pre-COVID-19 mean) were removed. Inclusion criteria (maximum of 25% missing data in the pre-COVID-19 period and a minimum of three months of data in the COVID-19 period per health area) led to different numbers of health areas kept for each indicator (Table S6 in the **Online Supplementary Document**).

To estimate how health service utilisation has changed at the beginning and during the COVID-19 period, we fitted a generalised additive model with first-order autoregressive correlation structure to each subregion using *R*, version 4.0.5 (R Core Team, Vienna, Austria) package ‘mgcv’ [45]:

γ_ij_ = negative binominal (μ_ij_)

μ_ij_ = off set(log (population)) + γ_1_ COVID period + γ_2_ COVID month + s (calendar month, bs = ’cc’, k = 12) + s (health area, centred month, bs = ’re’) + s (health area, bs = ’re’) + ε_ij_

Where γ_ij_ is the indicator of interest in health area *j* in month *i*. COVID period is a binary variable set to one if month *i* was during the COVID-19 period and zero otherwise. COVID month is a month since the beginning of COVID-19 period. S (calendar month, bs = ’cc’, k = 12 captures seasonality (shared at the subregion level). S (health area, centred month, bs = ’re’) captures health-area specific longer-term trend, modelled as a random effect, and s (health area, bs ‘re’) captures random intercept for each health area in the subregion. Details about sensitivity analysis are available in section 1.5.1 in the **Online Supplementary Document**. As measles and cholera cases were too sporadic to carry out interrupted time series analysis, we compared the average number of cases in the pre-COVID-19 and during the COVID-19 period. The COVID-19 period was from 1 April 2020 to 31 March 2021, and the pre-COVID-19 period was from 1 January 2017–31 March 2020.

We also calculated the difference with expected values in terms of the cumulative difference between the observed and expected number of consultations (by type) over the study period and the average monthly percent change in consultations for each month of the COVID-19 period at each facility (Section 1.5.2 in the **Online Supplementary Document**). Model diagnostics for all models are provided in Section 2.2.1 in the **Online Supplementary Document**.

#### Healthcare workers’ perceptions

One of the authors (HC) coded qualitative data with NVivo, and another researcher (CAL) reviewed it. The codebook included predefined codes corresponding to topics addressed in the interview guide and additional codes arising from the participants’ recounts. Answers were then organised into a matrix with one row per respondent and one code per column. Such framework analysis allowed for comparing responses by topic across participants [46].

#### Health care seeking behaviour

We reported each theme discussed during the focus groups into a saturation matrix, and we counted the number of mentions to identify the most common opinions. We then applied thematic analysis by summarising key findings per topic across focus groups. We prepared descriptive statistics (frequencies, means, proportions) through a weighted analysis of survey responses, disaggregated by age, sex, residence, and displacement status of respondents. We compared the proportions using paired-sample *t* tests. We considered *P*-values <0.05 statistically significant. We used R (packages ‘hypegrammR’ [47], ‘koboquest’ [48], and ‘surveyweights’ [49]) and Stata 14 for the quantitative analysis.

#### Ethics

We obtained the ethical clearance from Johns Hopkins Bloomberg School of Public Health’s Institutional Review Board (note 14 719 for components one, two, and three – non-human subject research; and note 15 447 for component four – human subject research). We received national authorisations from the ethical committees of the School of Public Health of the University of Kinshasa (letter ESP/CE/175/2020) and the University of Bukavu (letter UCB/CIES/NC/005/ 2021). Participation in the surveys and focus groups was voluntary, and each participant provided oral informed consent.

## RESULTS

### COVID-19 epidemiology

**Figure 1:**
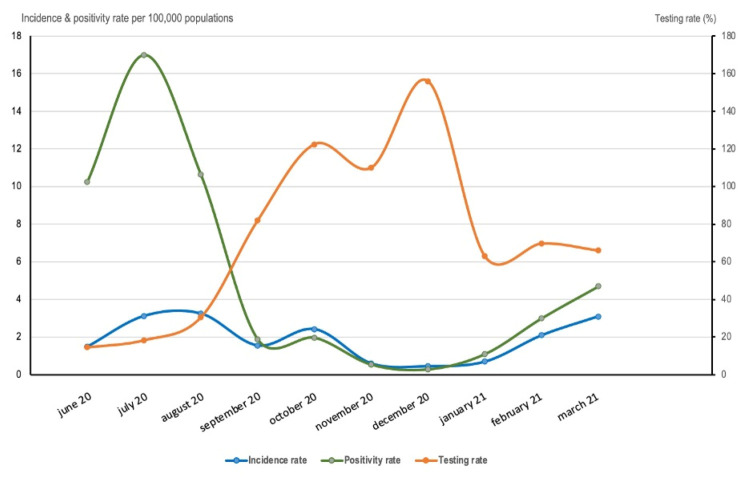
Trend of monthly testing, incidence, and positivity rate per 100 000 population, 1 June 2020 to 31 March 2021, North Kivu, DRC.

As only one COVID-19 case was recorded in Mweso health zone during the entire study period, we investigated the COVID-19 epidemiology at the provincial level (North Kivu). The number of confirmed cases in North Kivu augmented steadily, with a first wave in August 2020 and a second in March 2021. There were 2213 confirmed cases, corresponding to an incidence rate ratio (IRR) of 23.15 (95% CI = 22.18, 24.11) (**Table 1**). Most cases occurred among adults 18–59 years (mean age = 41.1 years), and less than 2% were reported among children under five. The highest incidence was in the older group (+60). Two-thirds of the cases were males. 244 deaths were recorded, corresponding to 11.1% case fatality rate (CFR; similar between men and women). The CFR ranged between 1.7% in May 2020 and 19.3% in March 2021. Older age and male sex were associated with higher odds of dying. 78 cases required hospitalisation (3.5%), all in the second wave (March 2021). 70 017 tests have been conducted during the study period, 1791 of which were positive. Testing rates increased in the last quarter of 2020, and the positivity rate was lowest during the same period (**Figure 1**). Tables S7–8 in the **Online Supplementary Document** provide descriptive results and mortality risk factors.

**Table 1:** Incidence, testing, and case fatality rates for the entire population and by age groups, North Kivu, DRC, 27 March 2020–31 March 2021*

		Age group in years		
**Items**	**Total**	**0–17**	**18–59**	**60+**
Line list data				
*Confirmed cases, n (%)*	2213	163 (7.5)	1635 (75.6)	364 (16.8)
*Population in thousands*	9560	4541 (47.5)	3718 (38.9)	449 (4.7)
*Incidence rate, 100 000/y (95% CI)*	23.15 (22.18, 24.11)	4.65 (4.02, 5.27)	42.68 (40.58, 44.77)	81.01 (72.69, 89.33)
*Confirmed deaths, n*	244	22	95	106
*Case fatality rates, % (95% CI)*	11.1 (9.8, 12.4)	13.5 (7.7, 16.4)	5.9 (4.8, 7.1)	29.2 (24.7, 34.0)
Testing data				
*Total number of tests done, n*	70 017			
*Positive test results, n*	1791			
*Testing rate, 100 000/y*	73.2			
*Positivity rate (%)*	2.6			

CI – confidence interval

*Source of population estimate: District Health Information System, Ministry of Health, DRC.

### Changes in health services utilisation

#### Outpatient consultations

**Figure 2:**
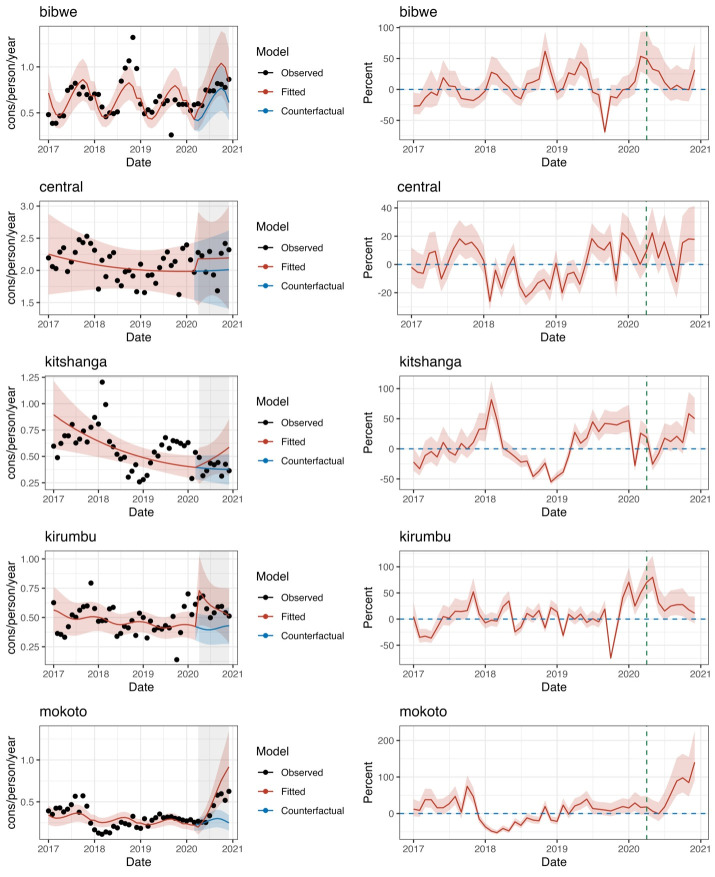
Mean health utilisation rate (observed values, fitted model and counterfactual) (left column) and percent monthly difference between expected and observed values, Mweso health zone, 1 January 2017 to 31 March 2021.

Increases were observed in most subregions when the pandemic began, from 5% in Kitshanga to 77% in Kirumbu (IRR = 1.775; 95% CI = 1.205, 2.614, *P* = 0.004). Slopes showed trends similar to pre-COVID-19, except for Mokoto where a 20% increase was seen (IRR = 1.198; 95% CI = 1.096, 1.308, *P* < 0.000). The cumulative number of outpatient department consultations was higher than expected in all subregions (from 4723 in Kitshanga to 23 692 in Kirumbu) (**Table 2**). The average monthly change ranged from 11% in the Central subregion to 58% in Mokoto (**Figure 2**).

**Table 2:** Interrupted time series results for the outcome of interest: immediate change, change in slope, cumulative difference, and percent monthly change, by subregion, Mweso health zone, 2017–21

Subregion	IRR (95% CI)	*P*-value	Cumulative difference, n (95% CI)	Average monthly change, % (95% CI)
**Health utilisation rate**				
Bibwe				
*Immediate effect*	1.291 (0856, 1.949)	0.244	6919 (3280, 10 430)	20 (11, 30)
*Change in slope*	1.008 (0.931, 1.091)	0.849		
Central				
*Immediate effect*	1.097 (0.806, 1.495)	0.556	23 692 (13 674, 34 720)	11 (6, 17)
*Change in slope*	0.999 (0.943, 1.059)	0.980		
Kitshanga				
*Immediate effect*	1.053 (0.716, 1.547)	0.794	4723 (3016, 6371)	18 (11, 26)
*Change in slope*	1.050 (0.977, 1.129)	0.183		
Kirumbu				
*Immediate effect*	1.775 (1.205, 2.614)*	0.004*	10 706 (8830, 12 793)	35 (27, 45)
*Change in slope*	0.952 (0.883, 1.026)	0.196		
Mokoto				
*Immediate effect*	0.870 (0.542, 1.396)	0.563	5860 (5076, 6731)	58 (45, 76)
*Change in slope*	1.198 (1.096, 1.308)*	0.000*		
**Malaria consultations**				
Bibwe				
*Immediate effect*	1.541 (1.166, 2.037)*	0.002*	6115 (5888, 6349)	95 (88, 102)
*Change in slope*	1.064 (1.007, 1.124)*	0.027*		
Central				
*Immediate effect*	0.925 (0.65, 1.317)	0.665	36 159 (28 621, 43 885)	28 (20, 36)
*Change in slope*	1.083 (1.013, 1.157)*	0.019*		
Kitshanga				
*Immediate effect*	0.564 (0.343, 0.926)*	0.024*	–5210 (–6610, –3701)	–27 (–33, –19)
*Change in slope*	1.082 (0.985, 1.188)	0.100		
Kirumbu				
*Immediate effect*	1.339 (0.91, 1.97)	0.139	–912 (–3034, 1210)	–2 (–9, –7)
*Change in slope*	0.926 (0.859, 0.997)*	0.041*		
Mokoto				
*Immediate effect*	0.631 (0.395, 1.009)	0.054	1070 (359, 1764)	11 (2, 21)
*Change in slope*	1.131 (1.037, 1.233)*	0.005*		
**Pneumonia consultations**				
Bibwe				
*Immediate effect*	1.110 (0.738, 1.671)	0.616	1898 (1359, 2421)	31 (21, 44)
*Change in slope*	1.082 (1.003, 1.167)*	0.042*		
Central				
*Immediate effect*	0.896 (0.626, 1.283)	0.548	962 (128, 1894)	7 (1, 14)
*Change in slope*	1.055 (0.984, 1.131)	0.134		
Kitshanga				
*Immediate effect*	0.773 (0.462, 1.293)	0.326	–479 (–892, –21]	–8 (–16, 1)
*Change in slope*	1.142 (1.037-1.257)*	0.007*		
Kirumbu				
*Immediate effect*	1.428 (0.889, 2.295)	0.141	1720 (1298, 2122)	33 (23, 44)
*Change in slope*	1.025 (0.935, 1.123)	0.598		
Mokoto				
*Immediate effect*	1.044 (0.556, 1.962)	0.893	2610 (2389, 2822)	153 (127, 185)
*Change in slope*	1.223 (1.09, 1.371)*	0.001*		
**ANC1**				
Bibwe				
*Immediate effect*	0.750 (0.576, 0.978)*	0.033*	–70 (–172, 34)	–2 (–6, 2)
*Change in slope*	1.079 (1.026, 1.135)*	0.003*		
Central				
*Immediate effect*	0.911 (0.716, 1.16)	0.451	–267 (–497, –10)	–4 (–7, 0)
*Change in slope*	1.010 (0.966, 1.057)	0.658		
Kitshanga				
*Immediate effect*	1.136 (0.899, 1.436)	0.285	284 (192, 381)	9 (6, 12)
*Change in slope*	0.986 (0.943, 1.032)	0.556		
Kirumbu				
*Immediate effect*	1.07 (0.874, 1.309)	0.513	346 (289, 405)	16 (13, 19)
*Change in slope*	1.021 (0.982, 1.062)	0.296		
Mokoto				
*Immediate effect*	1.210 (0.988, 1.482)	0.065	591 (550, 629)	44 (40, 48)
*Change in slope*	1.043 (1.005, 1.082)*	0.028*		
**Institutional deliveries**				
Bibwe				
*Immediate effect*	0.967 (0.731, 1.279)	0.815	144 (117, 172)	13 (11, 16)
*Change in slope*	1.056 (0.999, 1.117)	0.056		
Central				
*Immediate effect*	1.137 (0.894, 1.447)	0.295	142 (–171, 436)	3 (–2, 9)
*Change in slope*	0.971 (0.928, 1.016)	0.197		
Kitshanga				
*Immediate effect*	1.261 (0.858, 1.852)	0.238	601 (484, 713)	33 (25, 41)
*Change in slope*	1.025 (0.954, 1.102)	0.498		
Kirumbu				
*Immediate effect*	1.866 (1.385, 2.514)*	0.000*	484 (446, 521)	55 (49, 63)
*Change in slope*	0.950 (0.897, 1.006)	0.082		
Mokoto				
*Immediate effect*	1.105 (0.884, 1.382)	0.381	325 (303, 346)	43 (39, 47)
*Change in slope*	1.063 (1.02, 1.108)*	0.004*		
**Measles vaccination**				
Bibwe				
*Immediate effect*	0.972 (0.787, 1.201)	0.795	–125 (–191, –55)	–5 (–8, –2)
*Change in slope*	0.995 (0.956, 1.035)	0.790		
Central				
*Immediate effect*	0.925 (0.799, 1.071)	0.298	–246 (–312, –183)	–7 (–8, –5)
*Change in slope*	0.998 (0.971, 1.026)	0.896		
Kitshanga				
*Immediate effect*	0.912 (0.802, 1.038)	0.162	–508 (–569, –449)	–14 (–16, –13)
*Change in slope*	0.985 (0.962, 1.01)	0.236		
Kirumbu				
*Immediate effect*	0.995 (0.881, 1.124)	0.936	–92 (–138, –47)	–3 (–4, –2)
*Change in slope*	0.995 (0.972, 1.018)	0.666		
Mokoto				
*Immediate effect*	0.959 (0.86, 1.068)	0.446	21 (–2, 44)	1 (0, 3)
*Change in slope*	1.014 (0.993, 1.034)	0.187		

ANC1 – first antenatal care visit, CI – confidence interval, IRR – incidence rate ratio

*Statistically significant at *P* = 0.05.

#### Consultations for suspected malaria

Three subregions (Bibwe, Central, and Mokoto) reported 6115, 36 159, and 1070 consultations, respectively, over the COVID-19 period, likely because of the increasing slope over time (by 6, 8, and 13%) (**Table 2**). An immediate increase at the beginning of the pandemic was noted only in Bibwe (IRR = 1.541; 95% CI = 1.166, 2.037, *P* = 0.002; i.e. 54% increase). A 44% decrease (IRR = 0.564; 95% CI = 0.343, 0.926, *P* = 0.024) was reported in Kitshanga when the pandemic began, and a decreasing slope was seen in Kirumbu (IRR = 0.926; 95% CI = 0.859, 0.997, *P* = 0.041).

#### Consultations for mild pneumonia

**Figure 3:**
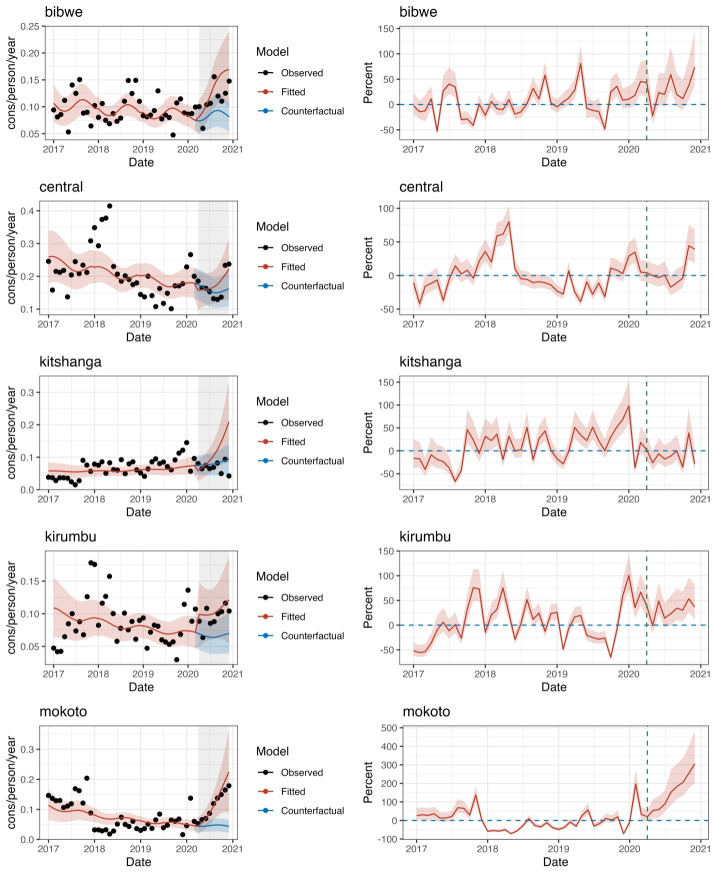
The mean utilisation rate for mild pneumonia (observed values, fitted model and counterfactual) (left column) and per cent monthly difference between expected and observed values, Mweso health zone, 1 January 2017 to 31 March 2021.

We found mixed results at the pandemic’s beginning. Two subregions experienced a decrease, and three others an increase. The change in slope was positive in all subregions (from 2.5% in Kirumbu to 22% in Mokoto). Results in Bibwe (IRR = 1.082; 95% CI = 1.003, 1.167, *P* = 0.042), Kitshanga (IRR = 1.142; 95% CI = 1.037, 1.257, *P* = 0.007), and Mokoto (IRR = 1.223; 95%CI = 1.09–1.371, *P* = 0.001) were statistically significant. The cumulative number of consultations was higher than expected in all but one subregion and ranged between 962 and 2610 consultations. The only subregion showing a decrease was Kitshanga (by 479 consultations) (**Figure 3**).

#### First antenatal care visit coverage

Kitshanga, Kirumbu, Mokoto reported an increase and Bibwe and Central a decrease. In Bibwe, IRR for immediate effect was 0.75 (95% CI = 0.576, 0.978, *P* = 0.033), corresponding to a 25% decrease when the pandemic began, and IRR for slope change was 1.079 (95% CI = 1.026, 1.135, *P* = 0.003), i.e. 8% monthly increase. Nevertheless, the cumulative number of antenatal care consultations was 70 less than expected. The positive balance in three subregions was likely due to a rise at the beginning of the pandemic (although none of the estimates is statistically significant) and an increase over time (except for Kitshanga). An increase in slope was seen in Mokoto (IRR = 1.043; 95% CI = 1.005, 1.082, *P* = 0.028).

#### Measles vaccination coverage

Although results were not statistically significant, vaccination coverage dropped in all subregions when the pandemic began (between 9% in Kitshanga and 1% in Kirumbu). All subregions but Mokoto had negative slope changes. The cumulative number of vaccine doses delivered was lower than expected in four subregions, ranging between 92 doses distributed in Kirumbu and 508 in Kitshanga. Monthly percent change ranged from –14–1%. Mokoto reported both a positive cumulative difference and a positive average monthly change.

#### Measles cases

The average weekly number of suspected measles cases was higher during the COVID-19 period than during the pre-COVID-19 period in 12 of the 21 health areas. A higher number of measles cases in the pre-COVID period was reported in two health areas (**Table 3**). The measles outbreak started in many health zones before the beginning of the COVID-19 pandemic. Nevertheless, the number of suspected measles cases occurring in the COVID-19 period was much higher than in the years before.

**Table 3:** Average weekly number of suspected measles cases in the pre-COVID-19 vs COVID-19 periods by health area and subregions, Mweso health zone, DRC, 1 January 2017–31 March 2021

Subregion and health area	Pre-COVID-19 period weekly number of cases (x̄)	COVID-19 period weekly number of cases (x̄)	Date of first week reporting cases
Bibwe	0.0645	6.2778	30 March 2020
*Bibwe*	0.0000	0.0000	
*Bweru*	0.0000	0.2188	25 May 2020
*Kivuye*			
Central			
*Bukama*	0.0066	1.6000	10 September 2018
*Bushanga*	0.0000	2.1429	6 April 2020
*Kalembe*	0.3986	2.2286	2 March 2020
*Kashuga*	0.2532	2.3636	13 March 2017
*Rugarama*	0.0068	0.0000	24 February 2020
Kirumbu			
*Busumba*	0.0000	0.0000	
*Kamonyi*	0.0000	0.2353	20 April 2020
*Katuna*	0.0000	0.0000	
*Kirumbu*	0.0129	0.1176	30 March 2020
Kitshanga			
*Burungu*	0.0000	0.5556	6 April 2020
*Kichanga*	0.0000	0.3429	22 June 2020
*Mwanja*	0.0000	0.0000	
*St Benoit*	0.1250	2.0833	10 February 2020
*Yopa*	0.0000	0.0000	
Mokoto			
*Kibarizo*	0.0000	0.0000	
*Mokoto*	0.0068	0.0000	13 January 2020
*Tambi*	0.0000	0.0000	
Mweso hospital	2.9677	5.3714	27 March 2017

x̄ – mean

#### Cholera cases

The average weekly number of cholera cases was higher in the pre-COVID period than in the COVID-19 period (Table S10 in the **Online Supplementary Document**). Sensitivity analysis is presented in Table S9 in the **Online Supplementary Document**.

#### Healthcare workers’ perceptions

Most HCWs reported a decrease in maternal, newborn and child health services. These were due to the absence of a technical and financial partner, stockouts of relevant commodities, fear of COVID-19 infections, unwillingness to comply with preventative measures, insecurity and population displacement: ‘The insecurity is the main cause of these changes, the activities could not be implemented as planned as the population is very mobile.’

Less affected were infectious disease services, referrals, and laboratory services. Community outreach services were reduced in frequency or size or completely interrupted, and non-communicable disease services were momentarily suspended in the centres that offered them. All respondents mentioned reduced availability of medicines due to border closures or lockdowns in March 2020. Most health facilities introduced infection prevention and control measures, although stockouts and limited access to water challenged their effectiveness. HCWs reported negative attitudes of the population regarding infection prevention and control measures: ‘Community members do not want to apply IPC measures as they do not believe at all in the existence of the COVID-19 pandemic.’

#### Community’s health care-seeking behaviour

Answers from the household surveys were provided for the first months of the COVID-19 pandemic (March–April 2020) and 30 days before data collection (October 2021) (Tables S11–13 in the **Online Supplementary Document**). Fewer households reported an illness event during the first months of the pandemic compared to October 2021 (23% vs 51%, *P* < 0.001). Among those who reported illnesses, almost all sought care in both periods (96% for March 2020 vs 93% for October 2021, *P* = 0.319) with little differences across sex or age groups, displacement status, and settings. Fever was the most common symptom, followed by cough, diarrhoea, chronic headaches, and breathing difficulty in both periods. The location where respondents sought care did not change in the two periods: the majority (about 60%) went to health centres, one-fifth to hospitals, and one-fifth to pharmacies. Most respondents who did not seek care at the time of data collection indicated the cost of treatment as the main barrier (81%), especially for younger adults and the elderly (both 100%) and for women (86%). When asked about routine child vaccinations, most respondents (79%) reported vaccinating their children during the first months of the COVID-19 restrictions. This was consistent across age groups, sex, residence, and displacement status. Among the reasons mentioned for not vaccinating children, interruption of services was the most common (22%), followed by fear of COVID-19 infection (21%). Access to care was reported to be difficult due to the associated financial burden, the limited availability of health facilities and the lack of medicines. Regarding changes in health care-seeking behaviour, all groups reported continuing to visit HCWs and health centres as they did before the pandemic. The cost of health services for people over five years old was the most common barrier to seeking treatment.

## DISCUSSION

We analysed complementary epidemiological and health data to form a comprehensive, although not exhaustive, picture of the situation in Mweso health zone, North Kivu province, during the first year of the COVID-19 pandemic. Two weeks after the first case of COVID-19 was detected (10 March 2020) [21], the DRC government announced a national state of emergency. COVID-19 response measures were instituted using lessons learned from previous Ebola virus disease outbreaks in DRC, including travel restrictions, lockdowns, widespread testing, quarantine, and community-based contact tracing [21]. In terms of strategy, the response was led by the Ministry of Health and organised around thematic pillars with partners’ support [22]. However, available financial and technical resources, including external partners’ presence in the country, were limited due to the disease being widespread nationally and globally. Questions remain as to how best to integrate a pillar response structure into the DRC health system to ensure the system identifies, responds, and adjusts following such a shock.

Demographically, cases in North Kivu were similar to COVID-19 cases worldwide, with males and adults being the most affected [50], children under five years being underrepresented, and the elderly overrepresented. Possibly, younger people were less open to testing, especially if asymptomatic or with mild symptoms. Most of the cases were identified in the provincial capital Goma, likely due to higher population density and testing capacity. Decentralised testing capacity remained limited within Mweso health zone. Rapid tests were available at the reference hospital and at a few health centres a few months into the pandemic, while polymerase chain reaction capacity was available only in Goma. It is difficult to estimate the level of underreporting as, to our knowledge, no seroprevalence survey was conducted in the Mweso health area. A seroprevalence survey took place in the two main markets in Goma in 2021 [12], reporting crude and adjusted seroprevalence rates of 70 and 98.8%. While informative, these results may not be extrapolated to a much more remote setting like Mweso. Ensuring that the testing capacity is quickly scaled up and available in different localities remains essential for future pandemic responses to understand the epidemiology and spread of diseases. As with other LMICs, equitable and decentralized access to testing capacity may not be possible or sufficient; in such cases, building upon existing sentinel site surveillance systems may generate crucial information across health areas to understand the disease epidemiology and to estimate more realistic case fatality rates. This may reduce concerns and encourage positive health-seeking behaviour in the population. At the same time, ensuring community acceptance remains instrumental in generating demand and using rapid tests.

The line list contained a few case management variables. Fewer cases in North Kivu (3.5%) needed hospitalisation compared to other countries (between 4–10%) [50], possibly because they were mild, as found in the seroprevalence survey in Goma [12]. Yet, given the high CFR (11%), underreporting and incomplete data cannot be excluded. The estimated CFR at the provincial level was higher than the national CFR (2.6%) [51] and similar to CFRs reported among hospitalised cases in Kinshasa [30,31] and in two other DRC provinces (Kwilu and North Ubangi) [52]. Although case underreporting likely inflated CFR, the mortality risk for severe cases was likely elevated due to the limited oxygen capacity and general accessibility to emergency services in remote areas of North Kivu, which were exacerbated by the multiple strikes of HCWs demanding safer working conditions.

The indirect effects of the pandemic on health care utilisation likely depended upon several factors, including adaptations in service delivery, national response measures and their enforcement, individual risk perception, trust in authorities and risk communication. Health services were not affected similarly. Routine immunisation services consistently experienced reductions in all subregions. Other studies at the national level and from Kinshasa reported mixed results [32–36]. This decrease is also not supported by the survey findings, which showed that caregivers continued to vaccinate children when COVID-19 restrictions were in place. Although fear of contracting COVID-19 has been found as one of the main reasons for reductions in routine vaccination uptake in low and middle-income countries [53], only a few survey respondents mentioned it. Recall and social desirability biases may explain this inconsistency. However, the lack of confirmed COVID-19 cases and the remoteness of the Mweso health zone may also have influenced the way communities perceived COVID-19. The number of measles cases increased in parallel to the drop in immunisation. While a measles epidemic has been ongoing in DRC since 2010 [54], Mweso reported more cases during the first year of the COVID-19 pandemic than in the three years prior, unlike at the national level, and despite at least two measles vaccination campaigns in 2020 [55]. This points to challenges in reaching children in remote and insecure areas. Advanced and decentralised vaccination strategies, for example, through mobile clinics or outreach activities, are an effective tool in the context of insecurity and can also reduce COVID-19 fears, as they limit population gathering at health facilities. The rise in infections is of particular concern given measles’ high mortality, especially among the high levels of acutely malnourished children in the health zone [56,57].

Utilisation of other health services increased in Mweso at the beginning of the pandemic and during its first year. This increase appears to be specific to Mweso, as other studies rather highlighted reductions at the national level [34,35], in Kinshasa [32], and in other countries in the region [58]. It is difficult to say whether these changes were related to the COVID-19 pandemic or due to population displacement in early 2020 following increased violence towards communities [59]. On the one side, this increase could be attributed to COVID-19 cases that were misdiagnosed as malaria or pneumonia, given that COVID-19 test availability was limited. On the other side, insecurity likely explains fluctuations in health care utilisation. The presence of armed groups, the fear they generate and the roadblocks they impose restrict access to farmland and health facilities, reducing the economic and physical capacity of the affected communities to access care. Health utilisation rates may have been erratic as people profited from relatively calm periods to access care for schedulable or planned interventions [60]. Accessing care for acute emergencies has proven more problematic, as movements were limited at various times due to insecurity. Therefore, the initiation of disease-specific policies and their implementation in areas affected by conflict and insecurity need further reflection on their direct and indirect consequences. While a few case studies investigated response strategies and mechanisms [61,62], implications at the community level are much less understood. Furthermore, data interpretation must be cautious due to the complex interaction between disease prevention and control measures and insecurity.

Findings from the survey also corroborate the quantitative results, as respondents continued to seek care in 2020 for similar symptoms and in the same locations as in late 2021. Financial constraints, not COVID-19, were reported as the main barriers to accessing health care. In DRC, out-of-pocket expenditures are the second largest source of health financing, following external aid, representing 40% of the total health expenditure [63] and 90% of household health expenditure. While health care is provided free for children under five years at health facilities supported by external partners, not all health facilities receive such support. During previous Ebola responses [64], free health care facilitated access to routine care. Yet, it was not utilised as a strategy in the COVID-19 pandemic response, likely because of the scale of the epidemic compared to Ebola. Achieving universal health care remains key, as ad hoc interventions temporally improve health access and outcomes of the populations but are not sustainable [65].

HCWs’ perceptions did not fully align with the quantitative results as they pointed to more interruptions or decreases, particularly in maternal and child health services and community outreach activities. Discrepancies may be due to recall bias and the overall complexity of perceiving trends that even out over time. While the quantitative estimation of changes in health service utilisation was conducted at one point (the beginning of the pandemic), over time, health care workers may more easily recall moments of very high or very low attendance, but not necessarily how they evolve. The lack or departure of external technical and financial partners was the predominant cause of such reductions, i.e. on the offer side. Budget shortages and conflicting donor priorities led to unstable partner presence and disrupted health care availability. The lack of a technical and financial partner is a well-known constraint in Eastern DRC, where externally supported health zones offer free and higher-quality services and have a more consistent drug supply. In contrast, health zones that only receive governmental support rely primarily on out-of-pocket expenses [60] and struggle with service provision. HCWs and community members reported drug stockouts, unfortunately also a known challenge. In 2017, antibiotics for children and paracetamol were available in only one-third of health facilities (nationwide) [66].

This work contributes to the limited evidence on COVID-19 from humanitarian settings. It provides insights that can be generalised to other remote conflict-affected areas such as the Central African Republic or South Sudan. These results are, however, likely less applicable to urban areas such as Goma or Bukavu, as population dynamics are quite different. Our analysis was limited by the variable availability in the COVID-19 line list. As no information about comorbidities was included, we could not identify non-demographic risk factors. Furthermore, we had to analyse COVID-19 data at the provincial level because only one COVID-19 case was recorded in Mweso health zone over the entire study period. The limited exposure to cases in Mweso may have influenced the community’s behaviour. Finally, in the interrupted time series analysis, we assumed that two parameters (an immediate and a long-term change) could capture fluctuations in health care utilisation during COVID-19. However, mechanisms through which COVID-19 affected health services may have been more complex due to disease dynamics and contextual challenges, and the model may not have captured more granular effects. The household survey was postponed due to armed violence and insecurity, which may have caused some recall bias in the respondents, possibly underreporting the effects of COVID-19 on health care-seeking behaviour. Consequently, the results need to be interpreted cautiously.

## CONCLUSIONS

The first year of the COVID-19 pandemic in Mweso health zone was characterised by low testing capacity and, likely, an underestimated number of reported COVID-19 infections. The increase in health care utilisation during the first year of the COVID-19 pandemic should be further explored to understand the role of factors unrelated to COVID-19. Insecurity, population displacement, and poverty remain major challenges to successfully implementing health programs and improving the population’s health outcomes. Furthermore, measles vaccination coverage dropped, which likely exacerbated the ongoing measles outbreak. Improved decentralised testing capacity will be crucial for future epidemics and enhanced efforts to maintain child vaccination coverage.

## Additional material


Online Supplementary Document
