# Supplementary material for: COVID-19 epidemiology, health services utilisation and health care seeking behaviour during the first year of the COVID-19 pandemic in Mweso health zone, Democratic Republic of Congo
Source: J Glob Health. 2024 Apr 26;14:05016. doi: 10.7189/jogh.14.05016 (PMC11047223; doi:10.7189/jogh.14.05016)
Supplement: Online Supplementary Document [file jogh-14-05016-s001.pdf]

Supplementary Material to the paper
"COVID-19 epidemiology, health services utilization and health care seeking behavior during the first year of the COVID-19 pandemic in Mweso health zone, Democratic Republic of Congo"

Table of Contents

1 Methods ... 3

1.1 COVID-19 line list completeness ... 3

1.2 Definition of indicators used in the health service utilization analysis ... 4

1.3 Health care workers profile ... 4

1.4 Primary data collection methods ... 4

1.5 Interrupted time series analysis of routine health data ... 7

1.5.1 Sensitivity analysis ... 7
1.5.2 Differences with expected values ... 8

2 Additional results ... 9

2.1 COVID-19 epidemiology ... 9

2.1.1 Descriptive statistics ... 9
2.1.2 Factors associated with mortality ... 9
2.1.3 Checking assumptions and model fit for logistic regression ... 9

2.2 Changes in health care utilization ... 11

2.2.1 Model diagnostic fits for Interrupted time series analysis. ... 11
2.2.2 Sensitivity analysis ... 14
2.2.3 Suspected cholera cases ... 15

2.3 Community perspective ... 17

List of Tables

Table S1: Completeness of variables in the COVID-19 line list ... 3
Table S2: Definitions of outcome indicators used in the analysis of changes in health service utilization ... 4
Table S3: Profile of health care workers interviewed, Mweso health zone, DRC ... 4
Table S4: Characteristics of focus group discussions' participants ... 5
Table S5: List of health areas by subregion, population size and live birth, Mweso health zone, DRC ... 7
Table S6: Number of health areas included in the analysis by outcome indicator, Mweso health zone, DRC ... 7
Table S7: Descriptive statistics of COVID-19 cases, March 27, 2020 to March 31, 2021, North Kivu, DRC ... 9
Table S8: Factors associated with mortality among confirmed COVID-19 cases, North Kivu, March 27, 2020 to March 31, 2021 ... 9

Table S9. ITS results for outcome of interest: immediate change (A), change in slope (B), cumulative difference (C) and percent monthly change (D), by subregion, Mweso health zone, 2017-2021. Model used has separate terms to capture potential changed in longer-term trend over years. 14
Table S10: Average weekly number of suspected cholera cases pre-COVID-19 vs COVID-19 period, by health area and subregion, Mweso health zone, January 1, 2017 to March 31, 2021, DRC 15
Table S11: Household survey results about health care seeking behavior: proportion of households reporting an illness event and proportion of households who sought care, at the beginning of the COVID-19 pandemic (March 2020) and the month before the survey (October 2021), Mweso health zone, DRC 17
Table S12: Household survey results about health care seeking behavior: proportion of households seeking care by health facility provider, at the beginning of the COVID-19 pandemic (March 2020) and the month before the survey (October 2021), Mweso health zone, DRC 18
Table S13: Household survey results about health care seeking behavior: proportion of households seeking care by reported symptoms, at the beginning of the COVID-19 pandemic (March 2020) and the month before the survey (October 2021), Mweso health zone, DRC 19
Table S14: Barriers for not seeking care during the month before data collection (Oct 2021), Mweso health zone, DRC 20

List of Figures

Figure S1: Map of assessed areas for household survey and FGD, Mweso health zone, DRC 6

1 Methods

1.1 COVID-19 line list completeness

Available individual-level variables included patient demographic information (i.d., age, sex, nationality, profession); location (i.d., health area, health district); test data (i.d., dates of sample collection, test, reason for testing); exposure risks (i.d., travels, contact with a confirmed case); case management (i.d., hospital status, site of treatment, date of discharge); and outcome of disease (i.d., recovery or death).

Within the 2,213 observations recorded, the percentage of completeness varied greatly across variables, ranging from 6.4% (site of treatment) to 100% (demographic variables). Table 4 summarizes the completeness of observations in the dataset. The variables concerning demographic features (age, sex, health area, health region, status) as well as date of testing and disease outcomes were the most complete. Profession and site of treatment were mainly missing (available for 6% of the cases). Information about travel were captured in the Comments section: we therefore created a categorical variable (Yes/No) for each case when information was available.

Table S1: Completeness of variables in the COVID-19 line list

Variable	Percentage
N of cases included in each data set	2,213
Unique Code	98.7%
Health area	83.3%
Health region	99.5%
Status (dead or survived)	99.9%
Sex	99.9%
Age	97.7%
Date of onset symptom	91.9%
Date of sample collection	99.8%
Date of investigation	99.6%
Date of PCR	99.6%
Outcomes	99.9%
Contact case	99.9%
Source of infection	99.9%
Identity of source of case	99.9%
Individual protection equipment	100%
Nationality	64.4%
Occupation	6.5%
Date of discharge	50.0%
Day of testing	99.9%
Comments	66.8%
Site of treatment	6.4%
Epidemiological week	99.9%
Week of discharge	99.9%

1.2 Definition of indicators used in the health service utilization analysis

Table S2: Definitions of outcome indicators used in the analysis of changes in health service utilization

Indicator	Numerator	Denominator
Health utilization rate	Number of new consultations, per month	Population in catchment area, divided by 12
Rate of consultations for suspected malaria	Number of suspected malaria cases, per month	Population in catchment area, divided by 12
Rate of consultations for mild pneumonia	Number of mild pneumonia cases, weekly	Population in catchment area, divided by 12
Antenatal Care 1 coverage (ANC1)	Number of ANC1 visits, per month	Number of estimated pregnant people in a year, divided by 12
Measles vaccine coverage	Number of measles-containing vaccine doses administered to children 0 – 11 months old. This includes doses administered via fixed, mobile, and advanced vaccination strategies.	Population 0 – 11 months old in catchment area, divided by 12
Number of measles cases	Number of measles cases, weekly	
Number of cholera case	Number of cholera cases, weekly	

1.3 Health care workers profile

Table S3: Profile of health care workers interviewed, Mweso health zone, DRC

Occupation	Number of respondents (n=39)
Registered nurses	15
Deputy nurses	5
Midwives	3
Nutritionists	3
Pharmacy attendants	2
Receptionists	2
Attending physicians	2
Assistant nurse	1
Birth attendant	1
Pharmacist	1
Pharmacy manager	1
Director of nursing	1
Chief of staff	1
Chief of emergency	1

1.4 Primary data collection methods

Both quantitative and qualitative components focused on health care seeking behavior at the beginning of the COVID-19 pandemic and how this changed over time (i.e., compared to the time of data collection).

Table S4: Characteristics of focus group discussions' participants

	Sex	Number of participants	Age category	Health zone	Settlement
**FGD 1**	Women	8	31 – 59	Mweso	Katuna
**FGD 2**	Men	8	18 – 30	Mweso	Katuna
**FGD 3**	Men	8	60 +	Mweso	Katuna
**FGD 4**	Men	9	18 – 30	Mweso	Bweru
**FGD 5**	Men	10	60 +	Mweso	Bweru
**FGD 6**	Men	10	31 – 59	Mweso	Bweru
**FGD 7**	Women	10	31 – 59	Mweso	Kitshanga
**FGD 8**	Women	9	18 – 30	Mweso	Kitshanga
**FGD 9**	Women	9	31 – 50	Mweso	Kitshanga
**FGD 10**	Women	9	18 – 30	Mweso	Mokoto
**FGD 11**	Women	10	60 +	Mweso	Mokoto
**FGD 12**	Men	10	31 – 59	Mweso	Mokoto

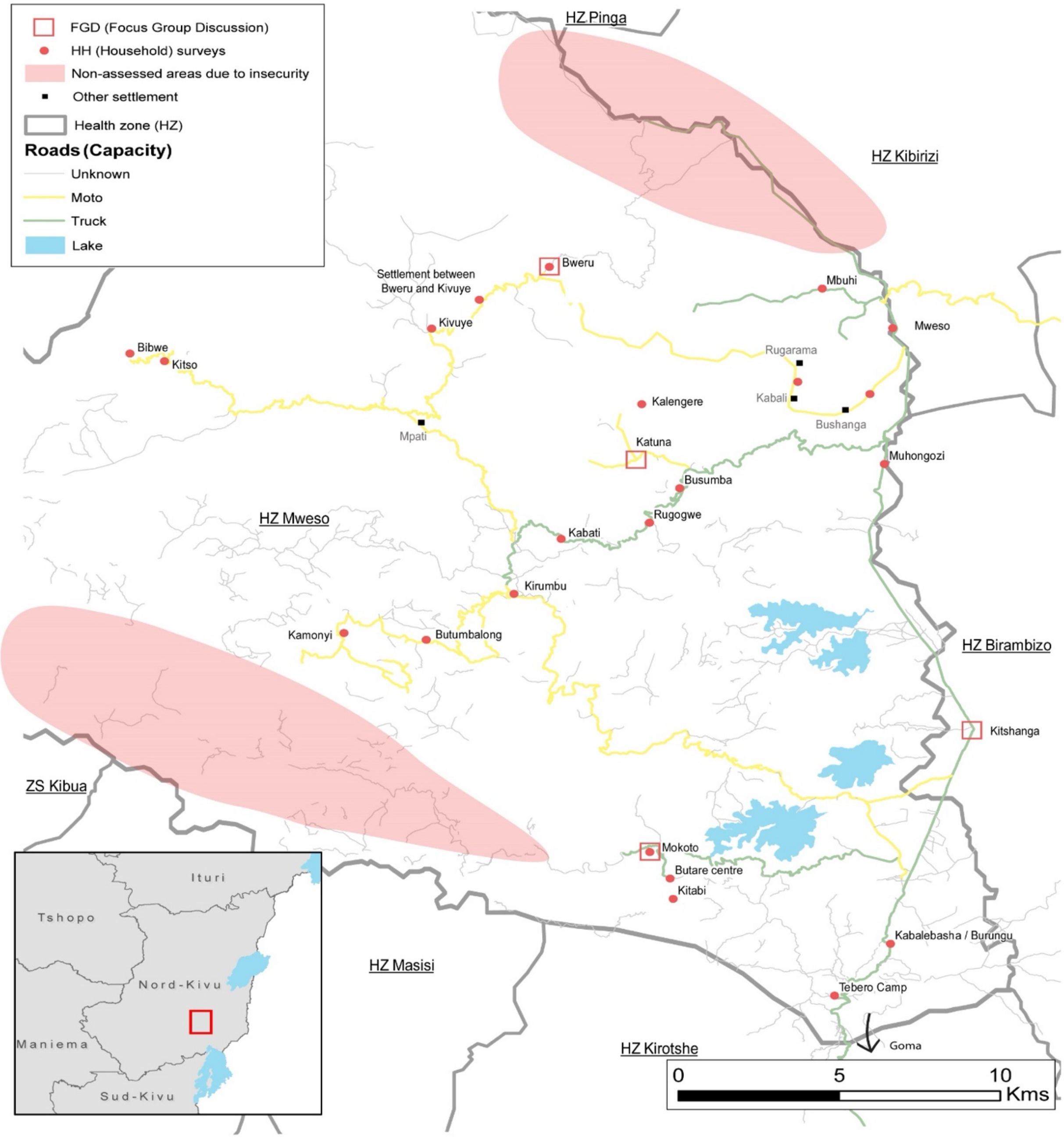

Figure S1: Map of assessed areas for household survey and FGD, Mweso health zone, DRC

1.5 Interrupted time series analysis of routine health data

Table S5: List of health areas by subregion, population size and live birth, Mweso health zone, DRC

Subregion	Health area	Population size (2017)	Estimated number of live births (2017)
Bibwe	Bibwe	27,570	1,268
	Bweru	12,202	561
	Kivuye	16,863	776
	Luhanga	7,200	331
Central	Bukama	15,299	704
	Bushanga	22,077	1,016
	Kalembe	21,661	996
	Kashuga	21,085	970
	Rugarama	20,921	962
Kirumbu	Busumba	16,968	781
	Kamonyi	16,150	743
	Katuna	11,566	532
	Kirumbu	25,375	1,167
	Lwama	10,285	473
Kitshanga	Burungu	22,305	1,026
	Kichanga	37,138	1,708
	Mwanja	7,421	341
	St Benoit	36,632	1,685
	Yopa	14,252	656
Mokoto	Kibarizo	15,557	716
	Mokoto	13,506	621
	Tambi	15,858	729

Table S6: Number of health areas included in the analysis by outcome indicator, Mweso health zone, DRC

Indicator	Subregion				
	Bibwe	Central	Kirumbu	Kitshanga	Mokoto
New consultations	4	5	5	5	3
Malaria	4	5	5	5	3
Diarrhea with dehydration	2	4	3	2	0
ANC1	4	5	5	5	3
Measles coverage	4	5	5	5	3
Mild pneumonia	4	5	5	5	3

1.5.1 Sensitivity analysis

As sensitivity analysis, we considered a model allowing for variation of trends at health area each year of the study period. This model takes the following form:

$$Y_{ij} = Negative\ Binomial\ (\mu_{ij})$$

$$\mu_{ij} = offset(\log(population)) + \gamma_1 COVID\ period + \gamma_2 COVID\ month$$
$$+ s(Calendar\ month, bs = \text{"cc"}, k = 12) + s(harea, Centered\ month, bs = \text{"}re\text{"})$$
$$+ s(harea, bs = \text{re"}) \text{+ s(harea, month\_2017, bs=}re\text{) + s(harea, month\_2018, bs=re)}$$
$$+ s(harea, month_{2019}, bs = re) + s(harea, month_{2020}, bs = re) + \epsilon_{ij}$$

Results of the sensitivity analysis are provided below.

1.5.2 Differences with expected values

A number of steps were required to estimate the difference between observed and expected cases:

- To estimate the counterfactual, or expected values during COVID-19 period, we first generated the expected value and standard error for each of the months in COVID-19 period using the fitted model, setting *COVID_period* and *COVID_month* to 0. We then drew 1,000 draws from a normal distribution with these parameters for each of the months in the study period.
- Prior to estimating difference between observed and counterfactual values, we imputed missing observed values. To do so, if a value was missing, we drew 1,000 draws from a normal distribution with parameters from the fitted model. If the value was not missing, then we used the observed value.
- To estimate cumulative difference for each subregion, we calculated the difference between observed and counterfactual value for each month for each health area and summed them across the entire COVID-19 period.
- To calculate monthly percent difference, for each of the 1,000 draws, we estimated the counterfactual cumulative number of consultations for each month, as well as the cumulative number of observed consultations for each month of the same period. We calculated percent difference between observed and counterfactual values for each month. To estimate median and 95% intervals for percent difference during COVID-19 period, across 1,000 draws, we obtained the median and lower and upper bounds of monthly percent differences by obtaining the 50th, 2.5th, and 97.5th quintiles for each month of the COVID-19 period.

2 Additional results

2.1 COVID-19 epidemiology

2.1.1 Descriptive statistics

Table S7: Descriptive statistics of COVID-19 cases, March 27, 2020 to March 31, 2021, North Kivu, DRC

	North Kivu province		
	**Total**	Female	Male
**Number of cases**	2,213	762 (35.31%)	1,398 (64.7%)
**Age** (mean + [range])	41.1 [0 to 125]		
Most affected age groups	18 to 50	18 to 50	18 to 50
**Disease outcome (N=2200)**			
Death	244 (11.1%)	78 (10.1%)	166 (11.7%)
Recovery	1,956 (88.9)	698 (89.9%)	1,255 (88.3%)
**Case management**			
Hospitalized	78 (3.5%)	29 (1.3%)	47 (2.1%)

2.1.2 Factors associated with mortality

Table S8: Factors associated with mortality among confirmed COVID-19 cases, North Kivu, March 27, 2020 to March 31, 2021

	Odds ratios	p-value	95% CI
**Age**			
0 – 17	2.59	<0.001	1.54 – 4.21
60+	6.55	<0.001	4.89 – 9.10
Ref: 18 – 59			
**Sex**			
Male	1.44	0.025	1.05 – 1.98
Ref: female			
**Health district**			
Goma	0.84	0.269	0.62 – 1.14
Ref: Other			

2.1.3 Checking assumptions and model fit for logistic regression

We assessed distribution of multicollinearity, presence of influence and outliers, and used Hosmer and Lemeshow goodness of fit test. Finally, we calculated the area under the curve. The outputs are presented below.

Multicollinearity
All of the VIF values are close to 1 (<1.1 for all three independent variables), so we don't have evidence for multicollinearity.

Hosmer-Lemeshow goodness of fit test
The p-value from this test is 0.9871; there is not enough evidence to reject the null hypothesis that the fit is OK.

AUC and ROC
AUC is 0.71. We would classify this as Acceptable discrimination.

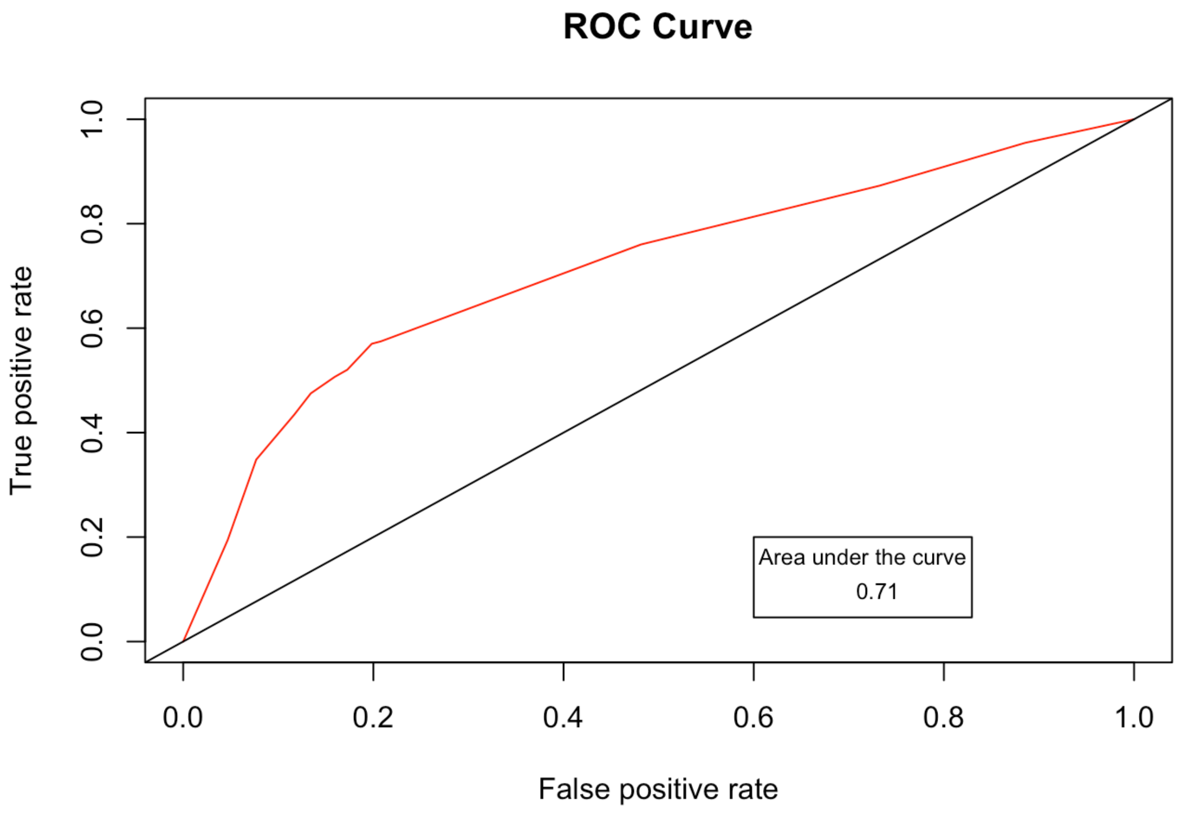

Outliers and influential observations
We identified four possible such observations. After removing these from data, we reran the regression. The change in the results is minimal.

	Odds ratios	Odds ratios (after dropping influential observations)
**Age**		
0 – 17	2.59 [1.54 – 4.2]	2.34 [1.36 – 3.86]
60+	6.55 [4.89 – 9.10]	6.65 [4.88 – 9.08]
Ref: 18 – 59		
**Sex**		
Male	1.44 [1.05 – 1.98]	1.45 [1.06 – 2.00]
Ref: female		
**Health district**		
Goma	0.84 [0.62 – 1.14]	0.82 [0.60 – 1.11]
Ref: Other		

Overall, the model fit seems reasonable, and we don't have strong evidence that any of the necessary assumptions are violated.

2.2 Changes in health care utilization

2.2.1 Model diagnostic fits for Interrupted time series analysis.

Model fit diagnostics, outpatient consulations

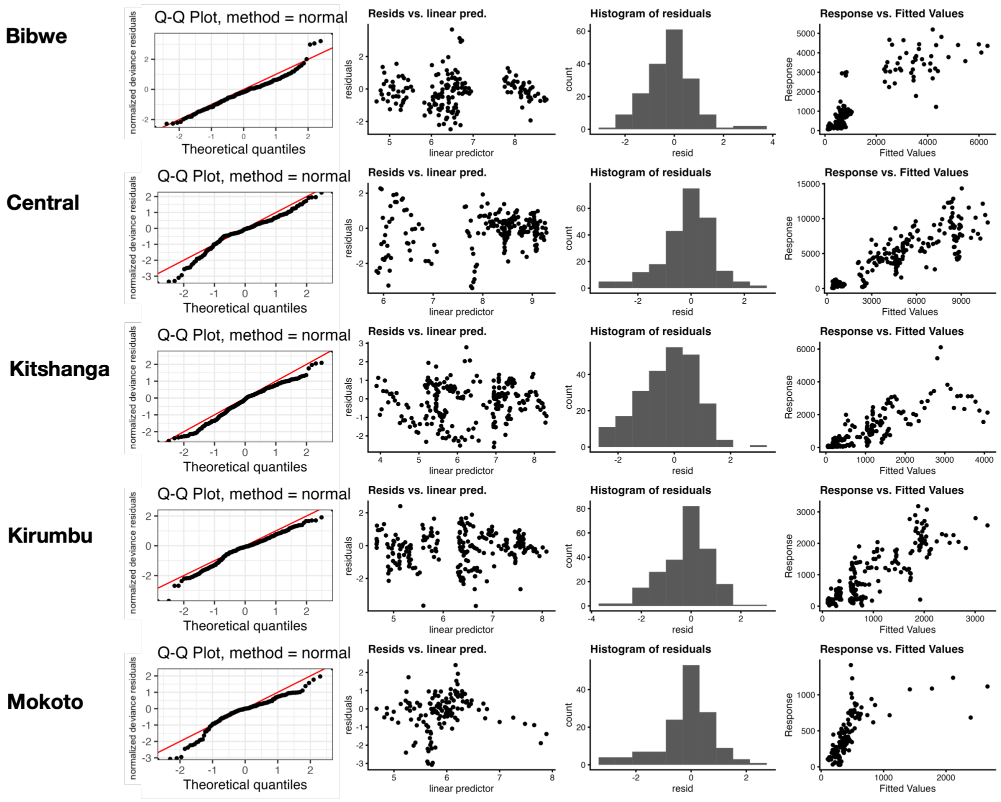

Model fit diagnostics, suscepted malaria cases

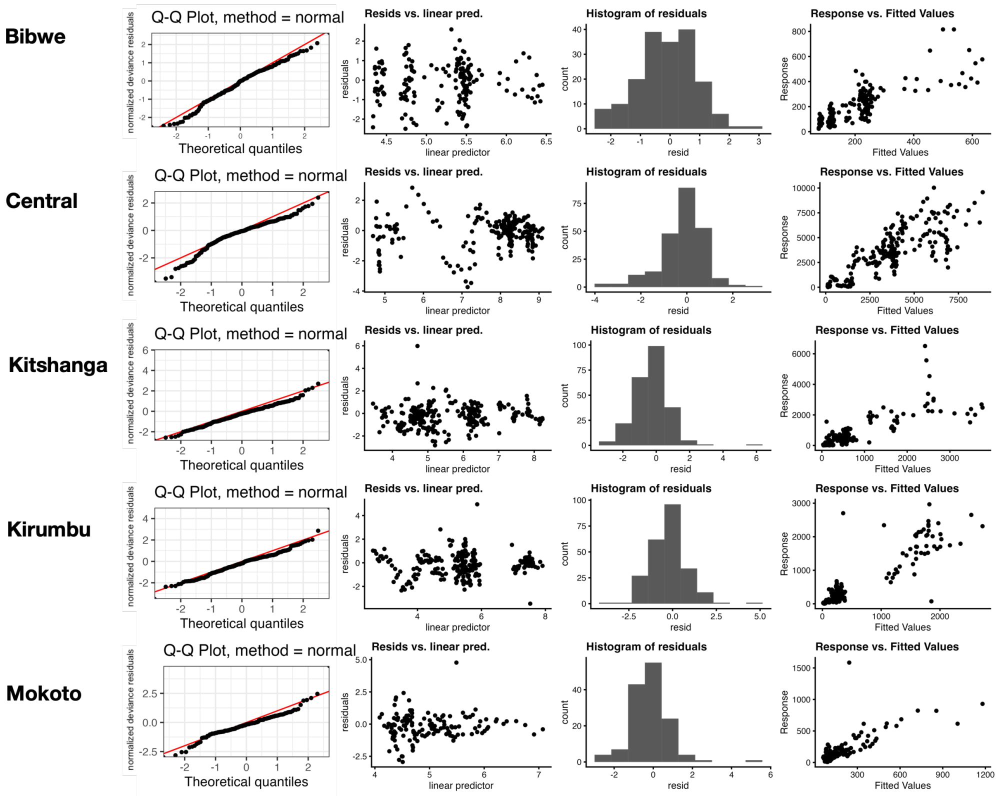

Model fit diagnostics, consultations for mild pneumonia

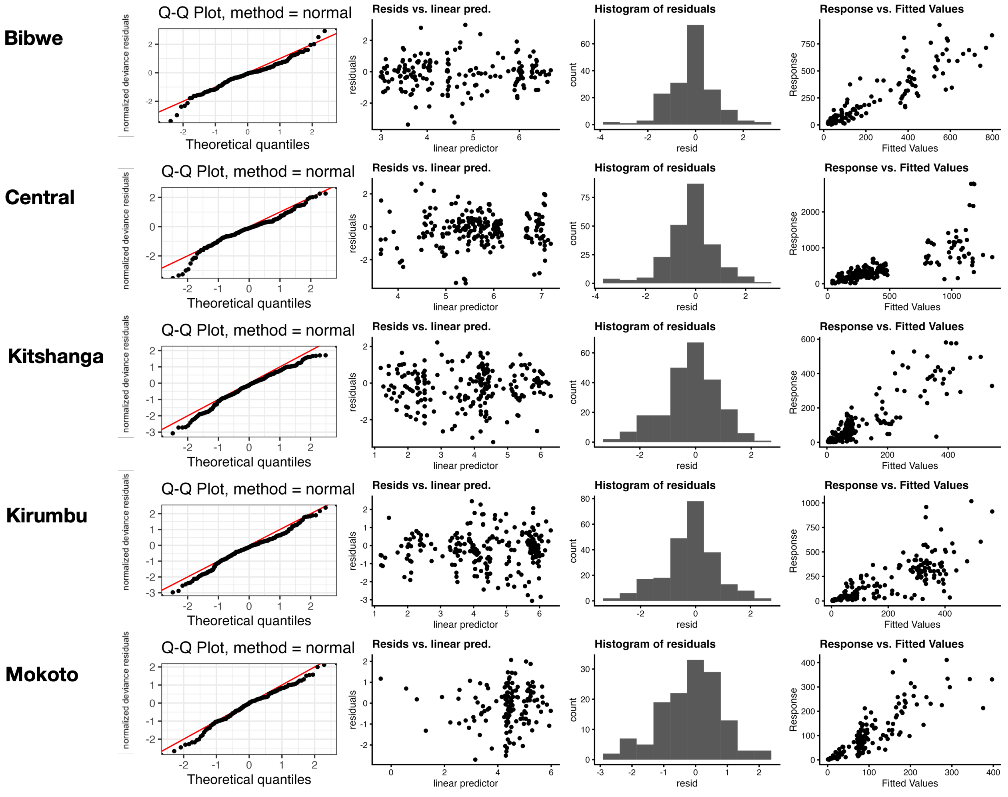

Model fit diagnostics, ANC1 coverage

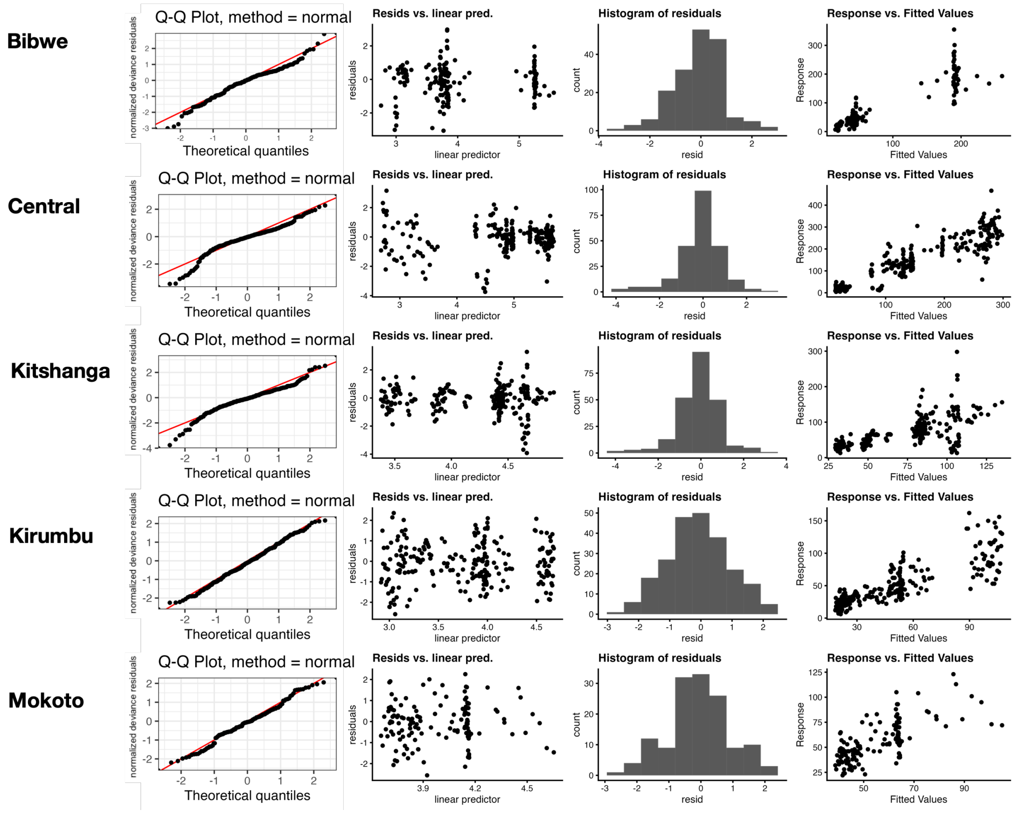

Model fit diagnostics, measles vaccination coverage

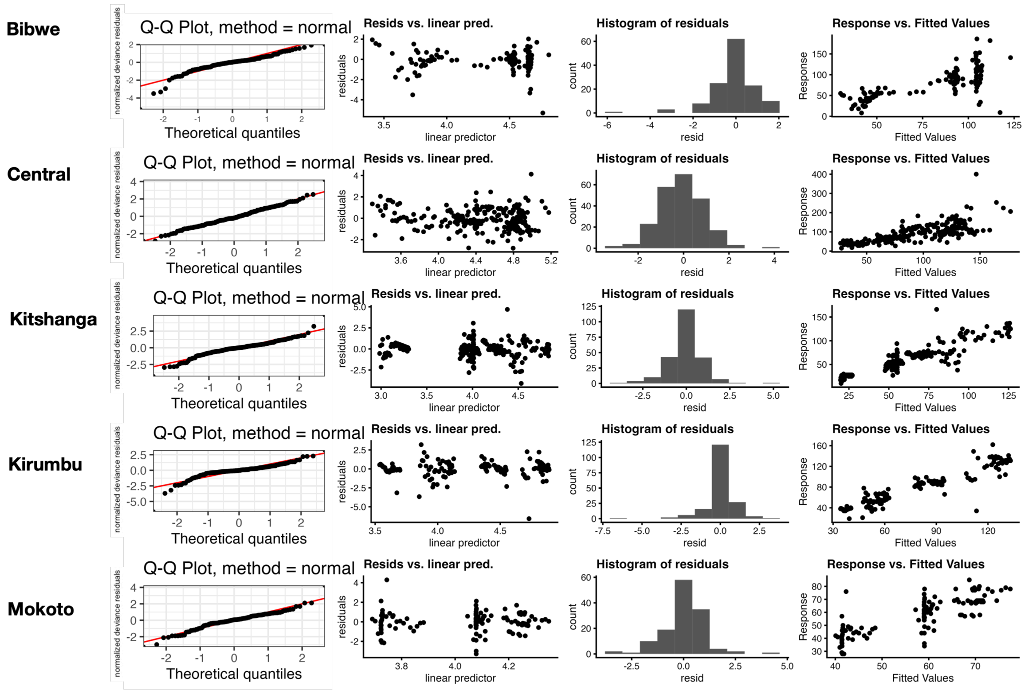

2.2.2 Sensitivity analysis

Table S9. ITS results for outcome of interest: immediate change (A), change in slope (B), cumulative difference (C) and percent monthly change (D), by subregion, Mweso health zone, 2017-2021. Model used has separate terms to capture potential changed in longer-term trend over years.

Subregion	Measures (A) and (B)	IRR [95%CI]	pvalue	C) Cumulative difference [CI]	D) Average monthly % change [CI]
**Health Utilization rate**					
Bibwe	Immediate effect	1.338 [0.976-1.835]	0.070	18,370 [15,669-21,023]	58 [45-74]
	Change in slope	1.056 [0.99-1.127]	0.098		
Central	Immediate effect	1.025 [0.74-1.419]	0.884	3,452 [-15,212-23,415]	3 [-5-13]
	Change in slope	0.994 [0.933-1.059]	0.850		
Kitshanga	Immediate effect	1.061 [0.7-1.61]	0.779	1,749 [-2,665-5,685]	9 [-6-35]
	Change in slope	1.006 [0.913-1.108]	0.912		
Kirumbu	Immediate effect	1.235 [0.779-1.959]	0.369	-15,042 [-24,447-(-6,127)]	-18 [-29-(-3)]
	Change in slope	**0.884 [0.791-0.988]**	**0.030**		
Mokoto	Immediate effect	0.832 [0.536-1.29]	0.410	6,427 [5,186-7,689]	76 [61-121]
	Change in slope	**1.173 [1.064-1.294]**	**0.001**		
**Malaria consultations**					
Bibwe	Immediate effect	**1.643 [1.234-2.186]**	**0.001**	6,778 [6,442-7,123]	119 [105-134]
	Change in slope	**1.071 [1.009-1.136]**	**0.023**		
Central	Immediate effect	0.783 [0.565-1.086[	0.142	16,107 [6,706-24,228]	11 [5-17]
	Change in slope	**1.078 [1.019-1.14]**	**0.009**		
Kitshanga	Immediate effect	0.731 [0.48-1.112]	0.143	-2,798 [-4,458-(-1,258)]	-16 [-25-(-2)]
	Change in slope	1.035 [0.949-1.13]	0.438		
Kirumbu	Immediate effect	1.05 [0.699-1.576]	0.815	-12,743 [-19,823-(-6,183)]	-27 [-37-(-8)]
	Change in slope	**0.895 [0.815-0.982]**	**0.020**		
Mokoto	Immediate effect	0.864 [0.602-1.239]	0.427	3,801 [3,326-4,247]	60 [47-73]
	Change in slope	**1.151 [1.084-1.221]**	**<0.001**		
**Pneumonia consultations**					
Bibwe	Immediate effect	1.207 [0.825-1.764]	0.333	3,227 [2,668-3,884]	76 [51-116]
	Change in slope	**1.094 [1.009-1.187]**	**0.029**		
Central	Immediate effect	0.821 [0.569-1.185]	0.292	-523 [-1,693-811]	-3 [-10-6]
	Change in slope	1.043 [0.974-1.118]	0.229		
Kitshanga	Immediate effect	0.837 [0.464-1.508]	0.554	-427 [-1,319-398]	-2 [-20-31]
	Change in slope	1.028 [0.892-1.185]	0.702		
Kirumbu	Immediate effect	1.112 [0.624-1.983]	0.718	-1,107 [-2,762-468]	-6 [-22-24]
	Change in slope	0.952 [0.826-1.097]	0.494		
Mokoto	Immediate effect	**0.261 [0.115-0.592]**	**0.001**	-67,817 [-109,383-(-35,115)]	-85 [-90-(-18)]
	Change in slope	0.787 [0.618-1.001]	0.051		
**ANC1**					
Bibwe	Immediate effect	0.954 [0.733-1.242]	0.725	719 [570-862]	43 [31-59]
	Change in slope	1.099 [1.038-1.163]	0.001		
Central	Immediate effect	1.043 [0.799-1.361]	0.757	690 [112-1,217]	14 [3-30]
	Change in slope	1.02 [0.936-1.112]	0.655		
Kitshanga	Immediate effect	1.084 [0.889-1.323]	0.425	50 [-94-204]	2 [-2-6]
	Change in slope	0.984 [0.946-1.023]	0.403		
Kirumbu	Immediate effect	0.923 [0.744-1.146]	0.470	81 [-57-202]	4 [-2-10]
	Change in slope	1.028 [0.979-1.079]	0.265		

Subregion	Measures (A) and (B)	IRR [95%CI]	pvalue	C) Cumulative difference [CI]	D) Average monthly % change [CI]
Mokoto	Immediate effect	1.214 [0.979-1.506]	0.078	595 [549-641]	45 [40-50]
	Change in slope	1.043 [1.006-1.081]	0.021		
**Institutional deliveries**					
Bibwe	Immediate effect	1.097 [0.819-1.47]	0.533	283 [193-368]	33 [21-49]
	Change in slope	1.042 [0.975-1.113]	0.223		
Central	Immediate effect	**1.282 [1.004-1.636]**	**0.046**	839 [-90-1,575]	22 [3-57]
	Change in slope	0.977 [0.887-1.075]	0.628		
Kitshanga	Immediate effect	1.111 [0.777-1.589]	0.563	240 [-44-510]	15 [1-35]
	Change in slope	1.002 [0.909-1.104]	0.976		
Kirumbu	Immediate effect	1.336 [0.969-1.841]	0.077	65 [-82-200]	6 [-4-21]
	Change in slope	0.93 [0.843-1.027]	0.150		
Mokoto	Immediate effect	1.056 [0.861-1.294]	0.604	290 [268-312]	37 [33-41]
	Change in slope	**1.063 [1.025-1.103]**	**0.001**		
**Measles vaccination**					
Bibwe	Immediate effect	0.972 [0.787-1.201]	0.795	-120 [-194-(-53)]	-5 [-8-(-2)]
	Change in slope	0.995 [0.956-1.035]	0.790		
Central	Immediate effect	**0.925 [0.799-1.071]**	**0.298**	-247 [-309-(-180)]	-7 [-8-(-5)]
	Change in slope	0.998 [0.971-1.026]	0.896		
Kitshanga	Immediate effect	0.912 [0.802-1.038]	0.162	-507 [-565-(-447)]	-14 [(-16)-(-13)]
	Change in slope	0.985 [0.962-1.01]	0.236		
Kirumbu	Immediate effect	0.995 [0.881-1.124]	0.936	-92 [-137-(-47)]	-3 [-4-(-2)]
	Change in slope	0.995 [0.972-1.018]	0.666		
Mokoto	Immediate effect	0.959 [0.86-1.068]	0.446	21 [-2-45]	1 [0-3]
	Change in slope	1.014 [0.993-1.034]	0.187		

2.2.3 Suspected cholera cases

Table S10: Average weekly number of suspected cholera cases pre-COVID-19 vs COVID-19 period, by health area and subregion, Mweso health zone, January 1, 2017 to March 31, 2021, DRC

Subregion	Health area	pre-COVID mean number of cholera cases (weekly)	COVID mean number of cholera cases (weekly)	date of first week reporting cases
Bibwe	Bibwe	0.0710	0.0000	2017-10-16
	Bweru	0.0133	0.0000	2018-02-05
	Kivuye	0.0000	0.0000	
Central	Bukama	0.0000	0.2857	2020-10-05
	Bushanga	0.0000	0.0000	
	Kalembe	0.0068	0.0000	2017-04-17
	Kashuga	0.3896	0.0000	2017-10-02
	Rugarama	0.0000	0.0000	
Kirumbu	Busumba	0.0000	0.0000	
	Kamonyi	0.8026	0.0000	2017-10-23
	Katuna	0.0000	0.0000	
	Kirumbu	0.1290	0.0000	2018-01-08
Kitshanga	Burungu	0.0000	0.0000	
	Kichanga	0.0408	0.0000	2019-05-20
	Mwanja	0.0000	0.0000	
	St Benoit	3.6645	3.3333	2017-12-04

Subregion	Health area	pre-COVID mean number of cholera cases (weekly)	COVID mean number of cholera cases (weekly)	date of first week reporting cases
	Yopa	0.0000	0.0000	
Mokoto	Kibarizo	0.2649	0.0000	2019-01-14
	Mokoto	0.0473	0.0000	2018-01-15
	Tambi	0.5948	0.1143	2017-10-16
	hgr mweso	3.1742	0.6000	2017-01-02

2.3 Community perspective

Table S11: Household survey results about health care seeking behavior: proportion of households reporting an illness event and proportion of households who sought care, at the beginning of the COVID-19 pandemic (March 2020) and the month before the survey (October 2021), Mweso health zone, DRC

	Proportion of HH who reported an illness event			Proportion of HH who sought care when ill		
	Mar-20	Oct-21	p-value	Mar-20	Oct 21	p-value
**Overall**	23% (20.04 - 26.53)	51% (46.85-54.52)	**<0.001**	96% (92.39 - 99.47)	93% (89.07 - 97.92)	0.319
**HoH's age**						
18-25	17% (11.87 - 22.47)	49% (41.46 - 55.51)	**<0.001**	96% (88.68 – 104.00)	96% (88.68 - 104.00)	1.00
26-35	27% (20.85 – 33.00)	48% (40.75 – 54.44)	**<0.001**	98% (93.45 – 102.00)	93% (86.06 -1.01)	0.1596
36-45	26% (17.53 - 34.32)	55% (46.03 - 65.08)	**<0.001**	95% (86.00 - 105.91)	86% (70.79 - 101.94)	0.1621
46+	24% (17.34 - 31.61)	55% (46.28 - 62.81)	**<0.001**	93% (82.69 - 103.03)	96% (89.10 - 103.76)	0.5732
**HoH's sex**						
Female	21% (16.75 - 24.35)	47% (41.89 - 51.27)	**<0.001**	96% (88.55 - 99.86)	97% (93.04 - 101.16)	0.3209
Male	29% (22.72 - 34.81)	59% (52.34 - 65.47)	**<0.001**	98% (94.43 - 101.86)	89% (80.23 – 97.55)	**0.0239**
**Displacement Status**						
Resident	22% (18.36 - 25.64)	51% (46.40 - 55.19)	**<0.001**	96% (91.12 - 99.89)	97% (92.81 - 100.45)	0.6573
Displaced	27% (20.34 - 34.44)	50% (42.41 - 58.23)	**<0.001**	97% (91.07 - 103.04)	85% (72.75 - 97.84)	**0.0437**
**Setting**						
Rural	25% (21.08 - 28.53)	52% (47.61 - 56.23)	**<0.001**	96% (92.47 - 99.91)	92% (87.22 - 97.54)	0.1583
Urban	18% (11.07 - 23.96)	46% (37.53 - 54.44)	**<0.001**	94% (82.72 - 106.17)	100% (100 - 100)	0.3313

Notes:
Mar 2020 = the first months of the COVID-19 pandemic
Oct 21 = 30 days before data collection
HH = Household
HoH = Head of Household

Table S12: Household survey results about health care seeking behavior: proportion of households seeking care by health facility provider, at the beginning of the COVID-19 pandemic (March 2020) and the month before the survey (October 2021), Mweso health zone, DRC

	Where did you seek care											
	Hospital			Health Center			Pharmacy			Traditional Healer		
	Mar-20	Oct-21	p-value	Mar-20	Oct-21	p-value	Mar-20	Oct-21	p-value	Mar-20	Oct-21	p-value
**Overall**	21% (13.33 -28.41)	18% (11.09 - 25.43)	0.4078	68% (59.16 - 76.49)	57% (47.32 - 65.72)	**0.0087**	20% (12.58-27.42)	17% (9.63 - 23.41)	0.4166	2% (-0.68 - 4.16)	3% (-0.35 -5.57)	0.3194
**HoH's age**												
18-25	31% (11.76 - 49.78)	31% (11.76 - 49.78)	1.00	69% (41.49 - 81.58)	62% (50.22 - 88.24)	0.3269	12% (-.16 - 24.70)	8% (-3.28 - 18.66)	0.6636	8% (-3.28 - 18.67)	8% (-3.28 - 18.67)	-
26-35	14% (3.16 - 24.74)	16% (4.78 - 27.78)	0.66	65% (50.28 - 79.96)	62% (37.96 - 69.02)	0.0959	23% (10.10 - 36.41)	19% (6.49 - 30.72)	0.486	0% (0 – 0)	0% (0 – 0)	-
36-45	21% (.86 - 41.24)	11% (-4.67 - 25.72)	0.1628	74% (51.87 - 95.49)	58% (33.45 - 82.34)	0.268	21% (0.86 - 41.24)	21% (0.86 - 41.24)	1.00	0% (0 – 0)	5.26% (-5.79 - 16.32)	0.3306
46+	22% (5.46 - 38.98)	15% (0.49 - 29.14)	0.3265	67% (35.52 - 75.59)	56% (47.66 - 85.67)	0.1846	22% (5.46 - 38.98)	19% (2.86 – 34.18)	0.6632	-	-	-
**HoH's sex**												
Female	25% (14.68 - 36.07)	19% (9.68 - 29.12)	0.2083	57% (44.54 - 68.89)	68% (57.26 - 80.06)	**0.0446**	21% (10.90 - 30.89)	16% (7.31 - 25.52)	0.4427	3% (1.19 - 7.17)	2% (1.197 - 7.17)	-
Male	15% (4.23 - 24.94)	17% (5.73 - 27.60)	0.5692	67% (52.83 - 80.49)	56% (41.69 - 70.81)	0.0959	19% (7.29 - 30.20)	16% (5.73 - 27.60)	0.7427	0% (0 – 0)	2% (-2.11 - 6.27)	0.3224
**Displacement Status**												
Resident	16% (8.32 - 24.24)	15% (7.39 - 22.84)	0.741	73% (63.71 - 82.80)	56% (45.10 - 66.52)	**0.0004**	21% (12.16 - 29.70)	16% (8.32 - 24.24)	0.3488	0%%	1% (-1.15 - 3.47)	0.3201
Displaced	34% (16.08 - 52.88)	28% (10.28 - 44.89)	0.3259	52% (39.55 - 77.69)	59% (32.38 - 71.07)	0.4238	17% (2.62 - 31.86)	17% (2.62 - 31.86)	1.00	7% (-2.91 - 16.71)	7% (-2.91 - 16.71)	-
**Setting**												
Rural	19% (10.68 - 26.43)	14% (9.82 - 25.23)	0.7075	67% (57.48 - 76.54)	58% (47.72 - 67.74)	**0.0383**	19% (10.68 - 26.43)	14% (7.31 - 21.55)	0.3738	2% (-0.82 - 4.94)	3% (-0.41 - 6.60)	0.3198
Urban	33% (9.21 - 57.46)	22% (0.95 - 43.50)	0.4299	72% (24.41 - 75.59)	50% (49.30 - 95.14)	0.1037	28% (4.86 - 50.69)	28% (4.86 - 50.69)	1.00	0% (0 – 0)	0% (0 – 0)	-

Notes:
Mar 2020 = the first months of the COVID-19 pandemic
Oct 21 = 30 days before data collection
HH = Household
HoH = Head of Household

Table S13: Household survey results about health care seeking behavior: proportion of households seeking care by reported symptoms, at the beginning of the COVID-19 pandemic (March 2020) and the month before the survey (October 2021), Mweso health zone, DRC

	Top five symptoms														
	Fever			Cough			Severe Diarrhea			Chronic Headaches			Breathing difficulty		
	Mar-20	Oct-21	p-value	Mar-20	Oct-21	p-value	Mar-20	Oct-21	p-value	Mar-20	Oct-21	p-value	Mar-20	Oct-21	p-value
**Overall**	73% (65.2 - 81.1)	63% (54.8 - 72.1)	0.0638	310% (21.9 - 38.3)	29% (21.1-37.4)	0.8702	17% (10.3-23.8)	11% (5.1-16.1)	0.0881	30% (21.9-38.3)	41% (31.9-49.5)	0.0523	15% (8.9-21.9)	11% (5.1-16.0)	0.2409
**HoH's age**															
18-25	85% (70.9 - 99.5)	81% (65.8 - 97.1)	0.713	15% (0.5 - 29.1)	22% (5.5-38.9)	0.4899	15% (0.4-29.1)	4% (-3.9-11.3)	0.1846	19% (2.9-34.2)	33% (14.3-52.3)	0.1612	19% (2.9-34.2)	11% (-1.6-23.8)	0.4246
26-35	74% (60.7 - 87.1)	63% (48.6 -77.5)	0.2292	30% (16.6- 44.3)	28% (14.7-41.8)	0.7429	20% (7.7-31.5)	13% (2.9-23.2)	0.2613	24% (11.1-36.7)	34% (20.5-49.1)	0.2292	13% (2.9-23.2)	7% (-.9-13.9)	0.3227
36-45	59% (36.8 - 81.4)	50% (27.3 - 72.7)	0.5396	32% (10.7- 52.9)	23% (3.7- 41.8)	0.4923	27% (7.1-47.5)	14% (-1.9-29.2)	0.2664	18% (0.7-35.7)	36% (14.5-58.2)	0.2133	23% (93.7-41.8)	14% (-1.9-29.2)	0.427
46+	71% (53.6 - 89.3)	57% (37.6 - 76.7)	0.161	28% (23.3- 62.4)	28% (23.3- 62.4)	1.00	7% (-3.0-17.3)	10% (-1.5-22.9)	0.5732	61% (41.4-79.9)	61% (41.4-79.9)	1.00	11% (-1.5-22.9)	14% (0.5-28.1)	0.6629
**HoH's sex**															
Female	78% (68.3 - 88.2)	65% (53.7 - 76.7)	0.06	32% (20.6- 43.2)	32% (20.6- 43.2)	1.000	27% (15.5-36.7)	14% (3.9-19.3)	**0.0114**	32% (20.6-43.2)	45% (32.9-56.9)	0.06	14% (5.9-23.0)	9% (3.9-19.4)	0.6408
Male	67% (53.7 - 79.7)	61% (47.7 - 74.5)	0.4964	28% (15.4- 40.1)	26% (13.9-37.9)	0.7844	6% (-7.6-11.9)	9% (1.3-17.3)	0.4193	28% (15.4-40.1)	35% (22.0-48.3)	0.3988	17% (6.4-26.9)	7% (1.3-17.3)	0.1592
**Displacement Status**															
Resident	73% (63.6 - 82.4)	65% (55.1 - 75.3)	0.1953	29% (19.6- 38.9)	33% (19.6- 38.9)	0.5804	13% (6.3-20.7)	8% (2.2-13.6)	0.1668	28% (18.6-37.6)	43% (32.2-53.2)	**0.0272**	15% (7.1-22.1)	12% (5.4-19.3)	0.64
Displaced	74% (57.9 - 89.2)	59% (41.4 - 76.3)	0.1689	32% (15.8-48.9)	21% (0.7- 34.9)	0.1604	26% (10.9-42.1)	18% (4.2-31.2)	0.3246	35% (18.4-52.2)	35% (18.4-52.2)	1.00	18% (4.2-31.2)	6% (-2.5-14.2)	0.1604
**Setting**															
Rural	72% (63.7 - 81.1)	66% (57.5 - 75.8)	0.3196	30% (21.6-39.4)	29% (19.8- 37.4)	0.7169	17% (9.8-24.5)	10% (3.8-15.2)	**0.045**	30% (20.7-38.4)	44% (34.2-53.5)	**0.0156**	17% (9.8-24.5)	9% (3.1-14.0)	**0.049**
Urban	78% (56.5 - 99.1)	44% (19.0 - 69.9)	**0.0096**	27% (4.9-50.7)	33% (9.2- 57.5)	0.7168	17% (-2.4-35.7)	17% (-2.4-35.7)	1.00	33% (9.2-57.5)	22% (0.9-43.5)	0.4299	6% (-6.2-17.3)	22% (0.9-43.5)	0.1872

Notes:
Mar 2020 = the first months of the COVID-19 pandemic
Oct 21 = 30 days before data collection
HH = Household
HoH = Head of Household

Table S14: Barriers for not seeking care during the month before data collection (Oct 2021), Mweso health zone, DRC

	Barriers for not seeking care*					
	Financial reasons (too expensive)	Illness not severe enough	Do not trust health care providers	Did not know how to access care	Security reasons (too dangerous)	Too far away
**Overall**	81%	10%	5%	5%	0%	0%
**Age of HoH**						
18-25	100%	0%	0%	0%	0%	0%
26-35	78%	11%	0%	11%	0%	0%
36-45	50%	25%	25%	0%	0%	0%
46+	100%	0%	0%	0%	0%	0%
**Gender of HoH**						
Female	86%	14%	0%	0%	0%	0%
Male	79%	7%	7%	7%	0%	0%
**Displacement Status**						
Non-displaced	83%	17%	8%	0%	0%	0%
Displaced	78%	0%	0%	11%	0%	0%
**Setting**						
Rural	81%	10%	5%	5%	0%	0%
Urban	----	----	----	----	----	----

Note: * Mulitple responses were possible
